# Supplementary material for: The influence of personality on psychological safety, the presence of stress and chosen professional roles in the healthcare environment
Source: PLoS One. 2023 Jun 5;18(6):e0286796. doi: 10.1371/journal.pone.0286796 (PMC10241408; doi:10.1371/journal.pone.0286796)
Supplement: S1 File — (DOCX) [file pone.0286796.s001.docx]

**Supporting Information 1: Topic Guide for Qualitative Interviews – Influence of Personality**

**THE INFLUENCE OF PERSONALITY ON PSYCHOLOGICAL SAFETY, THE PRESENCE OF STRESS AND CHOSEN PROFESSIONAL ROLES IN THE HEALTHCARE ENVIRONMENT**

**Version 0.2 26/07/19**

**IRAS Project ID: 263876**

**16PF Feedback and Qualitative Interview Topic Guide**

Welcome

*Introduction, overview of session*

- Welcome, introduction of researcher and project aims
- Overview of plan for study:
  - A short interview which will be recorded, explanation regarding data handling and anonymisation of transcripts.
  - 16PF feedback: Results presented as a written report, individual will take away copy to keep. Explanation that this feedback will not be recorded.
- Review of PIS and completion of written consent form
- “**Feel free to ask questions at any stage”**
- “**I may make notes so that we can return to a topic later in the interview”**

*Qualitative Interview*

**“We will now move on to a short interview to explore your opinions of stress within the working environment”**

**Perceived Stressors**

- What are your experiences of working under stress in the clinical department you work in?
- Experiences of stress at work – Do you find your working environment stressful? Is it always stressful?
- Do you enjoy the environment you work in?
- Coping strategies for stressful environments
- I am now going to read out a list of the 5 most common clinical stressors – can you please rank these in order of which you find most stressful?
  - High Workload
  - Patient Expectation
  - Conflict with Colleagues
  - Adequate Resources
  - Risk of Making a Mistake

**Personality**

- Do you see differences in personalities at work?
- How do you think your personality affects the way you manage stress? If at all?
  - Explore aspects of the participants personality that are both beneficial and detrimental
- Does this have an impact on the team?
  - Positive and negative experiences
  - How do you manage different personalities within the team?

**Psychological Safety**

- - Do you feel able to raise concerns within the team?
  - Do you think it is beneficial that all members of the team are able to raise concerns?
  - Please state whether you agree/ambivalent/disagree with the following statements, thinking about your experiences within your current workplace
    - **“If you make a mistake on this team, it is often held against you”**
    - **“Members of this team are able to bring up problems and tough issues”**
    - **“People on this team sometimes reject others for being different”**
    - **“It is safe to take a risk on this team”**
    - **“It is difficult to ask other members of this team for help”**
    - **“No one on this team would deliberately act in a way that undermines my efforts”**
    - **“Working with members of this team, my unique skills and talents are valued and utilised”**
