# Supplementary material for: The influence of personality on psychological safety, the presence of stress and chosen professional roles in the healthcare environment
Source: PLoS One. 2023 Jun 5;18(6):e0286796. doi: 10.1371/journal.pone.0286796 (PMC10241408; doi:10.1371/journal.pone.0286796)
Supplement: S3 File — (DOCX) [file pone.0286796.s003.docx]

**Supporting Information 3: Statistical Analyses**

Statistical Analysis of 16PF Traits – Emergency Department vs Critical Care

| **Primary Factor** | **t** | **df** | **Sig (2-tailed)** | **Mean Difference** | **Std. Error Difference** | **95% Confidence** | |
| --- | --- | --- | --- | --- | --- | --- | --- |
|  |  |  |  |  |  | **Lower** | **Upper** |
| Warmth | -0.686 | 61 | 0.495 | -0.279 | 0.407 | -1.094 | 0.535 |
| Reasoning | 1.192 | 61 | 0.238 | 0.547 | 0.458 | -0.37 | 1.463 |
| Emotional Stability | 2.275 | 61 | **0.026*** | 1.187 | 0.522 | 0.144 | 2.231 |
| Dominance | 2.454 | 61 | **0.017*** | 1.102 | 0.449 | 0.204 | 2 |
| Liveliness | 0.598 | 61 | 0.552 | 0.279 | 0.468 | -0.655 | 1.214 |
| Rule-Consciousness | -1.357 | 61 | 0.18 | -0.613 | 0.452 | -1.516 | 0.29 |
| Social Boldness | 0.91 | 61 | 0.366 | 0.445 | 0.489 | -0.532 | 1.421 |
| Sensitivity | -0.496 | 61 | 0.622 | -0.256 | 0.517 | -1.289 | 0.777 |
| Vigilance | -0.932 | 61 | 0.355 | -0.528 | 0.566 | -1.66 | 0.605 |
| Abstractedness | -1.893 | 61 | 0.063 | -0.968 | 0.511 | -1.99 | 0.055 |
| Privateness | -0.083 | 61 | 0.934 | -0.038 | 0.454 | -0.946 | 0.871 |
| Apprehensiveness | -2.309 | 61 | **0.024*** | -1.167 | 0.506 | -2.179 | -0.156 |
| Openness to Change | -0.119 | 61 | 0.906 | -0.052 | 0.439 | -0.931 | 0.826 |
| Self - Reliance | -0.497 | 61 | 0.621 | -0.255 | 0.513 | -1.281 | 0.771 |
| Perfectionism | -0.267 | 61 | 0.791 | -0.134 | 0.503 | -1.14 | 0.872 |
| Tension | 0.215 | 61 | 0.831 | 0.121 | 0.563 | -1.005 | 1.247 |

Spearman’s Rho Analysis assessing correlations between perceived clinical stressors and 16PF personality traits

|  |  | **Clinical Stressor** | | | | |
| --- | --- | --- | --- | --- | --- | --- |
| **16PF Primary Factor** |  | High Workload | Patient Expectation | Beds / Resources | Risk of Making a Mistake | Conflict with Colleague |
| Warmth | Correlation Coefficient | -0.083 | -0.043 | -0.009 | -0.045 | 0.005 |
|  | Sig. (2-tailed) | 0.537 | 0.749 | 0.947 | 0.735 | 0.972 |
| Reasoning | Correlation Coefficient | 0.197 | -0.069 | -0.122 | -0.071 | 0.171 |
|  | Sig. (2-tailed) | 0.139 | 0.604 | 0.363 | 0.597 | 0.2 |
| Emotional Stability | Correlation Coefficient | 0.206 | -0.229 | **-0.293^*^** | 0.189 | 0.239 |
|  | Sig. (2-tailed) | 0.121 | 0.083 | 0.026 | 0.154 | 0.071 |
| Dominance | Correlation Coefficient | 0.119 | -0.191 | -0.212 | 0.148 | 0.123 |
|  | Sig. (2-tailed) | 0.374 | 0.15 | 0.109 | 0.266 | 0.36 |
| Liveliness | Correlation Coefficient | 0.047 | **-0.286^*^** | -0.013 | -0.15 | 0.164 |
|  | Sig. (2-tailed) | 0.723 | 0.029 | 0.922 | 0.262 | 0.219 |
| Rule-Consciousness | Correlation Coefficient | 0.007 | -0.036 | 0.161 | 0.121 | **-0.263^*^** |
|  | Sig. (2-tailed) | 0.96 | 0.788 | 0.228 | 0.366 | 0.046 |
| Social Boldness | Correlation Coefficient | 0.083 | **-0.331^*^** | -0.011 | -0.108 | 0.256 |
|  | Sig. (2-tailed) | 0.535 | 0.011 | 0.935 | 0.421 | 0.053 |
| Sensitivity | Correlation Coefficient | -0.109 | 0.016 | -0.058 | 0.088 | 0.008 |
|  | Sig. (2-tailed) | 0.415 | 0.904 | 0.665 | 0.512 | 0.954 |
| Vigilance | Correlation Coefficient | -0.093 | -0.006 | 0.162 | -0.138 | -0.052 |
|  | Sig. (2-tailed) | 0.487 | 0.964 | 0.226 | 0.303 | 0.697 |
| Abstractedness | Correlation Coefficient | -0.16 | 0.236 | -0.09 | -0.059 | -0.021 |
|  | Sig. (2-tailed) | 0.231 | 0.075 | 0.503 | 0.661 | 0.878 |
| Privateness | Correlation Coefficient | 0.006 | 0.078 | 0.042 | 0.037 | -0.029 |
|  | Sig. (2-tailed) | 0.965 | 0.56 | 0.756 | 0.783 | 0.83 |
| Apprehension | Correlation Coefficient | -0.239 | 0.184 | 0.272^*^ | -0.185 | -0.197 |
|  | Sig. (2-tailed) | 0.071 | 0.166 | 0.039 | 0.164 | 0.138 |
| Openness to Change | Correlation Coefficient | -0.045 | 0.003 | -0.22 | -0.047 | 0.055 |
|  | Sig. (2-tailed) | 0.736 | 0.983 | 0.097 | 0.725 | 0.681 |
| Self - Reliance | Correlation Coefficient | 0.079 | 0.208 | -0.147 | 0.094 | -0.027 |
|  | Sig. (2-tailed) | 0.557 | 0.116 | 0.269 | 0.485 | 0.839 |
| Perfectionism | Correlation Coefficient | 0.106 | -0.045 | -0.014 | 0.155 | -0.031 |
|  | Sig. (2-tailed) | 0.43 | 0.738 | 0.914 | 0.246 | 0.82 |
| Tension | Correlation Coefficient | -0.005 | 0.082 | 0.054 | -0.048 | -0.074 |
|  | Sig. (2-tailed) | 0.971 | 0.541 | 0.685 | 0.721 | 0.582 |

Spearman’s Rho Correlation for Psychological safety and 16PF traits.

| **16PF Primary Factor** | **Item on Psychological Safety Assessment** | | | | | | | |
| --- | --- | --- | --- | --- | --- | --- | --- | --- |
|  | 1. Members of this team are able to bring up problems or tough issues 2. If you make a mistake on this team it is often held against you 3. People on this team sometimes reject others for being different 4. It is safe to take a risk on this team 5. It is difficult to ask other members of this team for help 6. No one on this team would deliberately act in a way that undermines my efforts 7. Working with members of this team, my unique skills and talents are valued and utilised | | | | | | | |
|  |  | 1 | 2 | 3 | 4 | 5 | 6 | 7 |
| Warmth | Correlation Coefficient | 0.177 | 0.052 | 0.113 | 0.093 | 0.05 | -0.007 | -0.106 |
|  | Sig. (2-tailed) | 0.184 | 0.7 | 0.399 | 0.487 | 0.708 | 0.961 | 0.430 |
| Reasoning | Correlation Coefficient | 0.117 | 0.078 | -0.21 | -0.076 | 0.201 | -0.004 | 0.236 |
|  | Sig. (2-tailed) | 0.382 | 0.559 | 0.114 | 0.569 | 0.131 | 0.978 | 0.074 |
| Emotional Stability | Correlation Coefficient | 0.027 | -0.057 | 0.063 | 0.154 | 0.201 | **0.272^*^** | 0.025 |
|  | Sig. (2-tailed) | 0.84 | 0.672 | 0.638 | 0.249 | 0.13 | **0.039** | 0.853 |
| Dominance | Correlation Coefficient | 0.124 | -0.183 | 0.191 | -0.1 | 0.17 | 0.205 | -0.131 |
|  | Sig. (2-tailed) | 0.352 | 0.17 | 0.15 | 0.456 | 0.203 | 0.122 | 0.327 |
| Liveliness | Correlation Coefficient | 0.23 | -0.222 | 0.094 | 0.16 | -0.101 | 0.072 | 0.161 |
|  | Sig. (2-tailed) | 0.082 | 0.094 | 0.485 | 0.23 | 0.451 | 0.592 | 0.226 |
| Rule-Consciousness | Correlation Coefficient | 0.034 | 0.04 | 0.23 | 0.012 | 0.205 | 0.058 | -0.08 |
|  | Sig. (2-tailed) | 0.798 | 0.765 | 0.082 | 0.928 | 0.123 | 0.666 | 0.549 |
| Social Boldness | Correlation Coefficient | 0.15 | -0.215 | 0.116 | 0.091 | -0.047 | 0.026 | 0.037 |
|  | Sig. (2-tailed) | 0.26 | 0.105 | 0.385 | 0.499 | 0.724 | 0.845 | 0.782 |
| Sensitivity | Correlation Coefficient | 0.055 | -0.13 | 0.004 | 0.034 | -0.237 | -0.037 | 0.193 |
|  | Sig. (2-tailed) | 0.684 | 0.333 | 0.978 | 0.798 | 0.074 | 0.785 | 0.146 |
| Vigilance | Correlation Coefficient | 0.046 | -0.093 | -0.042 | -0.026 | -0.042 | -0.049 | -0.101 |
|  | Sig. (2-tailed) | 0.733 | 0.487 | 0.757 | 0.848 | 0.753 | 0.718 | 0.452 |
| Abstractedness | Correlation Coefficient | 0.027 | -0.111 | **-0.277^*^** | -0.002 | -0.176 | -0.217 | 0.082 |
|  | Sig. (2-tailed) | 0.84 | 0.408 | **0.035** | 0.987 | 0.185 | 0.102 | 0.54 |
| Privateness | Correlation Coefficient | 0.031 | 0.142 | 0.069 | -0.019 | -0.056 | -0.019 | -0.112 |
|  | Sig. (2-tailed) | 0.817 | 0.289 | 0.605 | 0.89 | 0.678 | 0.889 | 0.403 |
| Apprehension | Correlation Coefficient | -0.06 | 0.079 | -0.136 | -0.059 | -0.098 | -0.245 | -0.028 |
|  | Sig. (2-tailed) | 0.654 | 0.556 | 0.308 | 0.662 | 0.465 | 0.064 | 0.835 |
| Openness to Change | Correlation Coefficient | 0.051 | -0.02 | -0.016 | 0.217 | -0.057 | -0.229 | -0.02 |
|  | Sig. (2-tailed) | 0.703 | 0.881 | 0.905 | 0.101 | 0.672 | 0.084 | 0.88 |
| Self - Reliance | Correlation Coefficient | 0.046 | -0.018 | 0.049 | -0.018 | -0.197 | 0.054 | -0.004 |
|  | Sig. (2-tailed) | 0.73 | 0.896 | 0.714 | 0.892 | 0.138 | 0.689 | 0.978 |
| Perfectionism | Correlation Coefficient | 0.105 | 0.065 | 0.021 | 0.139 | 0.127 | 0.216 | 0.09 |
|  | Sig. (2-tailed) | 0.432 | 0.626 | 0.876 | 0.297 | 0.34 | 0.104 | 0.499 |
| Tension | Correlation Coefficient | 0.048 | 0.032 | -0.123 | -0.122 | -0.043 | -0.247 | -0.129 |
|  | Sig. (2-tailed) | 0.722 | 0.81 | 0.359 | 0.363 | 0.749 | 0.062 | 0.334 |
