# Supplementary material for: The influence of personality on psychological safety, the presence of stress and chosen professional roles in the healthcare environment
Source: PLoS One. 2023 Jun 5;18(6):e0286796. doi: 10.1371/journal.pone.0286796 (PMC10241408; doi:10.1371/journal.pone.0286796)
Supplement: S4 File — (DOCX) [file pone.0286796.s004.docx]

**Supporting Information 4: Supporting Qualitative Data for the Influence of Personality Thematic Framework**

| **Theme** | **Subtheme** | **Supporting Data** |
| --- | --- | --- |
| Teamwork | “Fitting in” | “I think that’s a bit of the thing about if you don’t fit the mould, then it’s harder to fit you into the work environment that everyone else is heading towards” 0035, Consultant, Emergency Department  “And one of the reasons that I joined is personality based, because I consider my personality a bit outside the rest of the team and I’m a bit different with them. In terms of pressure I’m more chill, I’m more cheerful.” 0049 Staff Nurse, Critical Care  “I won’t take everything too personally, I just accept that people deal with different things very differently and they are very different. And sometimes everyone has a bad day and everyone has a good day. That doesn’t make them a bad person.” 0055, Sister, Critical Care  “You do also get some of the quieter type as well, but it’s usually the quite confident people that you find are A&E nurses, because you have to be able to think on your feet quiet quickly.” 0037, Staff Nurse, Emergency Department  “I think being a certain type of personality helps for the ED, personally.” 0069, Consultant, Emergency Department  “I think different personalities, because it’s such a big team, but there’s a lot of people, relatively short attention span, variety, a bit of excitement, teamwork.” 0035, Consultant, Emergency Department |
|  | Impact on team dynamics | “Personalities always affect the teamwork, unless you realise that everyone is very different and you accept the fact that everyone is different. Sometimes, people clash, but that’s not because anyone wants to clash, it’s just because we are different.” 0055, Sister, Critical Care  “It’s a totally different dynamic depending on who’s together, and it’s not even always about, sometimes, but it’s not necessarily about individual personalities, it’s about the people that get together. So, if you get five people that really like to complain together, then it’s just a really dreadful shift. Or five people that are a little bit lazier than everybody else, then it’s like, can you just do your work and focus on what you’re supposed to be doing?” 0083, Staff Nurse, Emergency Department.  “Yes. I do feel that way. I think there is a bit of… I mean, it’s a biased view, because I might feel some personalities more soothing as compared to what somebody else might think. So it’s quite subjective, but that’s true. I mean, if I know that I’m working with certain people for the entire week, and if I know who they are and how they think, that always puts me at ease.” 0087, Consultant, Critical Care  “it’s just sometimes it depends on certain people, and their personality, or sometimes just a certain constellation that stresses us so much, that we all lose our temper a little bit.” 0092, Consultant, Critical Care.  “But it’s like little dream teams that I know if they’re all going to be on shift together put them all in the same place those patients will be fine. But even if they’re not particularly friends they’ll all work together they will get in touch. And if you put them without a little support system they don’t work quite as well even if they’re great independently.” 0068, Senior Staff Nurse, Critical Care  “I’ll offer help for people that you know will take it, but you know some people, they just like to do everything themselves. I think it’s just getting to know people and knowing when they’re having an off day and if and what you can do to help them.” 0086, Staff Nurse, Critical Care  “there are definitely a few personality clashes within the team, but we’re quite a small, close-knit team.” 0056, Staff Nurse, Critical Care  “I think personality always influences with how we decide and how we think, how to handle any particular situation.” 0087, Consultant, Critical Care  “some people are quite… Pushy is not the right word, they are quite upfront and they won’t hesitate to express their views, and they can get things done very easily.” 0087, Consultant, Critical Care  “They get really stressed very easily. I think in that situation, once someone who’s a lead, you can visibly see them stressed, it has a really negative effect, a knock on effect of everyone else. So instead of positively influencing people, it’s actually quite negative.” 0089, Staff Nurse, Critical Care |
|  | Conflict | “But it kind of makes it a little bit harder as well, because you do get personalities and I think you get big personalities in ICU anyway. But you can get a little bit of eye-rolling and what have you and this kind of thing going on.” 0080, Staff Nurse, Critical Care  “It’s a personality thing sometimes I think and when you’re new to an environment people’s attitude towards you can kind of be funny.” 0080 Staff Nurse, Critical Care  “The only issues I had maybe was personality clashes, but I guess that’s normal in any workplace.” 0086, Staff Nurse, Critical Care  “But I’ve had a few tiffs with people, but like you said, it’s been more personality wise.” 0086, Staff Nurse, Critical Care  “I think that’s probably because of the perhaps overly perfectionist attitude that I have, where I don’t particularly like to be in a situation where there’s conflict, which is perhaps unrealistic.” 0075, Physiotherapist, Critical Care |
|  | Psychological Safety | “I think often it’s a choice of who you would go to and that’s probably in terms of personality and how you get on with some people.” 0042, Junior Doctor, Emergency Department  “I usually tend to say what I think.” 0047, Physiotherapist, Critical Care Department  “It has to be with personality in the way that some people will be more comfortable naturally to speak up. But to establish a normal speaking relationship and instead of using it as a concern, just friendly talk about it. Different people will approach to that thing differently, but it is one of the most important things in intensive care to speak up.” 0049, Staff Nurse, Critical Care  “I’m finding that it’s difficult to ask questions. I feel like I shouldn’t be the one asking questions.” 0070, Junior Doctor, Critical Care  “I think because I’m normally so chilled as well, and that’s another reason why people come to you and unload as well, so I think a double-edged sword, and it’s useful in clinical practice.” 0072, Senior Staff Nurse, Critical Care  “I think, most of the time, you can raise concerns with, as expected, variability between who’s on and the different personalities. So, I suppose it’s, with certain people, raising it in the right way. Yes.” 0075, Physiotherapist, Critical Care  “But then again, it kind of does depend on the personalities around you, if you do have a hectic day and you do need help from the person in the bay next to you. I’ve had shifts where people have been brilliant, but I’ve also had shifts where you were just ignored and can you get somebody else to do it or you know.” 0080, Junior Doctor, Emergency Department  “Well, we’d hope they’d be able to, but sometimes they don’t. And that’s maybe personality.” 0084, Staff Nurse, Critical Care  “Although I mean that’s personality, as well, if someone doesn’t always want to ask for help. I can think I’m not going to ask for help until I’m really at my limit and then I thought I actually should have asked for help before” 0084, Staff Nurse, Critical Care  “I’m not the person who will challenge the senior people… But it depends on the consultant as well, it depends on the personality.” 0090, Senior Staff Nurse, Critical Care |
| Individual Personality Traits | Impact of one individual on the environment | “So it’s the people who are resistant to change that really frustrates me, and there are a few people in my department and within my consultant body who are like that.” 0038, Consultant, Emergency Department  “But I do know, just that, depending on who’s on the unit or who is in charge of a particular scenario, obviously you’ll see things work different.” 0050, Senior Staff Nurse, Critical Care  “My colleagues, particularly my senior colleagues, i.e. my consultant that I’m on with, can change the level of stress.” 0079, Junior Doctor, Critical Care  “From the consultants on to the registrar that you’re with or if you’re on your own, and your juniors, and the nurse in charge of [unclear] can totally, totally change your day.” 0079, Junior Doctor, Critical Care  “Whether or not a day is stressful can also depend on who you’re buddied up with more than anything. And I’m buddied up with this person; it’s going to be a long 12 hour shift. But the same goes in the other direction and you’re great, I’ve got so-and-so, this is going to be fun.” 0081, Junior Staff Nurse, Critical Care  “Anybody who’s got a stressed personality I feel can influence outcomes because we all need each other within the facility.” 0091, Staff Nurse, Critical Care  “So those are few people that were noticeably very stressed, and then other people would get stressed around them. And normally I’m the opposite and it’s okay, but there were parts when I’d get a bit like that.” 0072, Senior Staff Nurse, Critical Care  “if the department is a lot busier and you are working with people that are likely to get stressed a bit more easily, then, yes, that would be, definitely an influence.” 0040, Staff Nurse, Emergency Department  “But also it’s the different personality types. But also just the day and how people are feeling because you can have the same team on one day and it works really well, and another day it just doesn’t work so well for whatever reason. There’s not a particular kind of trigger point, but I guess if one person’s in a bad mood it makes everyone else in to a bad mood.” 0046, Sister, Critical Care  “So you’ll know when a particular consultant and a particular nurse in charge [unclear], it’s not going to be smooth sailing.” 0069, Consultant, Emergency Department.  “Our one person who can totally change the mood of the scenario, nobody’s on edge not doing their best, because if you’re relaxed, most of the time you actually get the job done better. But one person can put… That doesn’t have to be a consultant, it can be, not even the nurse in charge, it can just be one person in that team that it’ll just put the whole team on edge. And that can make a whole difference in the scenario really.” 0050, Senior Staff Nurse, Critical Care  “And actually, I think sometimes it is useful to have those people on the team. But yes, there are some people that are just generally, quite, potentially, quite negative and that… You could feel that atmosphere depending on who was on.” 0051, Sister, Critical Care  “One person in particular, when she is in charge on shift we all sigh a breath of relief. And we just sit back in our metaphorical deck chairs and we’re just like, yes she’s on we don’t need to worry. She’s got it. She’s got it. She’s just amazing.” 0071, Senior Staff Nurse, Critical Care  “I think if you’re a bit flappy or disorganised and all over the place it rubs off on other people, I definitely noticed that.” 0072, Sister, Critical Care  “if you’ve got someone that’s calm and like it’s going to be fine, you’re going to be okay, I’m here if you need anything just let me know, that tends to make them feel instantly relaxed for the shift.” 0072, Sister, Critical Care  “Like even today, we had a lovely consultant on, very chilled in the morning, bit more tense in the afternoon and literally just the whole department felt that way. And that, you know, it was a lot busier in the afternoon, so there’s a lot more pressures, but I think a calm consultant has a huge influence on the whole department.” 0074, Junior Doctor, Emergency Department  “Because you sometimes know if you’ve got one particular consultant in charge or reg in charge, you just know it’s going to be a bit more of a stressful day.” 0078, Junior Doctor, Emergency Department  “Before you even get to work, you just know maybe they’re just a little bit less experienced and a bit more anxious about the whole thing, or they just like to be a bit more in control or sometimes they just, I don’t know. But yes, you can just… And that’s probably just their personality type.” 0078, Junior Doctor, Emergency Department  “I do think it makes a difference. I really do. It’s the ripple effect, all of us feel it.” 0085, Staff Nurse, Critical Care  “With some members of the team, it was fine, but there was particular people that would make the day difficult…. It’s their personality.” 0054, Senior Staff Nurse, Critical Care  “it depends what their personality is, their leadership style, their experience. That has a huge impact on what the outcome of the shift will be and how we will handle all the stresses that will come up throughout the shift.” 0076, Junior Doctor, Emergency Department  “Well, I think in ICU, because there are so many things involved, anyone is capable of disrupting that rhythm. It could be a consultant from another team who’s pushing for beds, or it could be a family member, it could be a patient himself, or anyone really speaking.” 0087, Consultant, Critical Care  “Yes, if there’s a very proactive registrar or SHO and they come round and explain without... Even if they just pop their heads in every now and again just to say like, is everything all right? I think they can have quite a big influence on making it a bit less manic.” 0088, Staff Nurse, Critical Care  “I’d agree that the consultant and nurse-in-charge are the most important. We have a slightly more complex, sort of, nursing, well complex system in that there are two consultants, so both consultants obviously can affect it, and also we have a floor coordinator who oversees the whole unit, but then a nurse-in-charge on each side.” 0092, Consultant, Critical Care  “Yes, those kind of personality affects me, it makes it more stressful.” 0090, Staff Nurse, Critical Care  “I’ve watched other nurses, for example, who aren’t really getting anything done because they’re stressed or panicking” 0084, Staff Nurse, Critical Care |
|  | Awareness of impact of own personality on team | “No, my personality or how they interpret my personality has been a challenge for me and for them, but I manage to fit in.” 0049 Staff Nurse, Critical Care  “I think I have a personality which itself… I mean, I feel that everybody gets that I’m a very approachable person, and I’ve always had people, and trainees especially, coming to me and sharing what bothers them, even during [the] pandemic. And I’m always very supportive of them, and I will do what I can to help them.” 0087, Consultant, Critical Care.  “It just takes one person to lift the mood, to lighten the mood. I feel that because I would be quite goofy and stuff, and I would probably tell a few jokes on the ward round, just to lighten things.” 0089, Senior Staff Nurse, Critical Care  “And there were times when I’d probably snap or I’d hurry people on and say actually no I don’t have time for this, I’ll speak to you later on or whatever. And that’s just not how I normally would communicate with people, and I’d notice that I was getting stressed at that stage.” 0072 Senior Staff Nurse, Critical Care.  “I’m quite level and externally people really don’t see much difference. I’m quite calm.” 0035, Consultant, Emergency Department  “So I think I very much had a let’s just get on with it type of attitude. Which I know can be quite annoying for some people, because it’s, oh actually I don’t want to get on with it, this is a horrible situation, so stop being so perky.” 0051, Sister, Critical Care  “And then I definitely noticed I’m normally quite calm. I don’t shout at people. I don’t tend to get that stressed, and I’m normally quite relaxed.” 0072, Sister, Critical Care  “I mean hopefully, but when I’m in a cardiac arrest, if I’m the nurse in charge, I’ll step back and let people do what they want and try and direct people to do certain things if they’re not being done. And try and get people involved as much as possible. So hopefully that’ll make it relaxed because I’ve been there forever.” 0050, Senior Staff Nurse, Critical Care |
|  | Suitability for role | “in A&E you get rewarded for being confident, because it makes the seniors less stressed.” 0034, Junior Doctor, Emergency Department  “I’m very extrovert anyway, so that’s why I can’t have a proper career anywhere. So yes, I’m very outspoken” 0076 Junior Doctor, Emergency Department  “I wouldn’t consider myself a very shy person, which helps but I think it comes under that whole fear of me not doing enough and not doing a good enough job.” 0062 Physiotherapist, Critical Care  “on a baseline personality, I’m quite confident.” 0092, Consultant Critical Care  “I quite like high pressure, quick decisions, moving fast.” 0041, Junior Doctor, Emergency Department  “You do also get some of the quieter type as well, but it’s usually the quite confident people that you find are A&E nurses, because you have to be able to think on your feet quiet quickly.” 0037, Staff Nurse, Emergency Department  “My personality type is I’m not comfortable with a long waiting time and I’m not comfortable feeling out of control so I feel best when it’s a manageable list of patients.” 0039, Junior Doctor, Emergency Department  “I quite like high pressure, quick decisions, moving fast. I would say I’ve got a four-hour attention span so that’s why I’m good at ED. But then I also quite like having a chat to people. I quite like meeting people. I’m quite personable that way. So I’ve got this sort of best blend of both of them.” 0041, Junior Doctor, Emergency Department  “I’m quite good at forgetting about things when I need to work.” 0046 Senior Staff Nurse, Critical Care  “I don’t know if personality and mindset are the same thing, I don’t know, but the way I look at things, I’m like if I get stressed I can’t do my job best that I could do it.” 0046 Staff Nurse, Critical Care  “I think that’s part of the reason I’m in emergency medicine is, it’s not about a long lead in and prepping the surgery or anything like that. You’ve got to respond to the thing in the moment. And so, if there’s something about the ability to handle uncertainty.” 0052, Consultant, Emergency Medicine  “I’m fairy chilled, I am not a control freak by any stretch of the imagination. So not knowing what’s coming into my day is actually good for me, because I like variety. I’m not interested in doing the ward round on Monday morning for two hours and then clinic, that’s my idea of hell.” 0052, Consultant, Emergency Medicine  “there’s no point in my becoming upset about things that I can’t control. That’s an aspect of my character, which I think I’m sometimes grateful for, and sometimes ashamed of, that I will just accept what’s happening and get on.” 0059, Senior Staff Nurse, Critical Care  “I think I’m quite an organised person normally. So, I have an order where I do things. So, that helped. I’m very clean as well. My bed space was always quite clean.” 0065, Staff Nurse, Critical Care  “I think being a certain type of personality helps for the ED, personally……… I think I’m very much a kind of doer and I make… Any decision’s better than nothing. So I think that suits emergency medicine quite well. 0069, Consultant, Emergency Department  “I think generally ITU nurses tend to be the type A personality who expects everything to be done to perfection” 0046, Senior Staff Nurse, Critical Care  “I’m confident to work within my competency scope and I’m very confident to ask when I need the help.” 0091, Senior Staff Nurse, Critical Care  “So, I always try and make a conscious effort to keep myself calm and then I feel like I can do my own job better. So, I imagine it must be personality.” 0048, Senior Staff Nurse, Critical Care  “I’ve just never had a real problem with containing any kind of stress level or feeling like it’s, all getting on top of me. So maybe that is personality. Yes, it probably is a bit of personality.” 0084, Staff Nurse, Critical Care  “So, I think part of ICU is being very methodical, and detailed, and not missing anything, and taking your time, and doing it to the best of your ability. But, then, I stress about my ward round taking too long or I stress about the fact that I still write everything on my sheet, whereas the other consultants breeze around with no sheet, stuff like that.” 0077, Consultant, Critical Care  “Some people saying wow, you generally stay quite relaxed, and things like that” 0040, Staff Nurse, Emergency Care  “I’ve always been quite an anxious person and I care too much I think about patients and about things.” 0047, Physiotherapist, Critical Care  “Not giving yourself enough credit and I think my personality is I’m a bit of a people pleaser and need to know that I’m doing the right thing and doing the best that I can and I think it made me stressed because I wasn’t getting enough feedback.” 0062, Physiotherapist, Critical Care  “People tell me that I’m calmer, very calm. I don’t talk much. Even though I am stressed inside, I don’t tend to show it outside.” 0090, Staff Nurse Critical Care |
|  | Individual response to stress | “There are people that are stressed quite easily” 0040, Staff Nurse, Emergency Department  “I wouldn’t describe myself as someone that gets easily stressed. I get frustrated quite easily, but I don’t feel stressed.” 0034, Junior Doctor, Emergency Department  “They’re very risk-averse and worried about the fact that there could be a diagnosis that they’ve missed, even if it’s not an emergency diagnosis.” 0035, Consultant, Emergency Department  “There are people that are stressed quite easily” 0040, Staff Nurse, Emergency Department  “Yes it is because I see a lot of people worry, and I know that it factors in many people’s lives.” 0071, Sister, Critical Care  “I think some people get very caught up if they are stressed and they let it get to them a lot. Firstly at work, when something’s not going their way, I think some people can find it quite difficult to keep plodding on, you know?” 0088, Staff Nurse, Critical Care  “I like to feel in control and I can tend to be anxious. So for me, I try to anticipate anything that will make me anxious and prevent it rather than let it happen and deal with it.” 0039, Junior Doctor, Emergency Department  “Although I’m a worrier, I’m not a massively stressful… I certainly don’t outwardly protract the stress.” 0051, Sister, Critical Care  “I think, in general, I’m not really a stressy person, until it gets to the nitty-gritty, the last minute. It takes quite a fair bit to get me really stressed, in general, and then, obviously, working in that environment, you learn very quickly that you just need to take a deep breath a lot of the time and relax, otherwise it all goes wrong. Because it’s organised chaos at the end of the day, I think that’s the big thing.” 0060, Junior Doctor, Emergency Department  “I think my personality has sort of contributed to maybe being seen as a bit more vocal, but also going with the flow when things are quiet and trying to just do other things to prepare for when it will get busy.” 0081, Staff Nurse, Critical Care  “I think some people perhaps aren’t that bothered about whether they get everything done or not and some people yes perhaps they’re just a little bit more chilled out than me, a little bit more willing to go with the flow” 0064, Junior Doctor, Critical Care  “I think specifically with stressful things I do see a big difference in the way that people handle things. I think some people are straight in there, they’re fine, life is fine we’ll move on I just keep going, no problem. Other people really need to go off the floor and take a break, have a breather and we’ll send you away for half an hour. And I don’t think that is even relative to the level of experience.” 0068, Staff Nurse, Critical Care  “I know it’s done in an educational manner but if it’s something that if you’re someone who naturally would feel bad about those sorts of things rather than being able to rationalise it, you can take that very badly.” 0042, Junior Doctor, Emergency Department  “I’m convinced that some people blossom in under stress and are able to reach out and become much more team players. And then others retreat back into command control mode and I think that varies also with your team.” 0069, Consultant, Emergency Department  “I think though some people thrive on stress, that’s how they get through life, it’s they bumble through stress.” 0071, Senior Staff Nurse, Critical Care  “I think it’s the personality of the individuals and their perception of stress and their ability to cope and manage their own stress. And how they can first understand their stress and how they can do sensible things to try and support it so that they can have a better day and that everyone else is safer.” 0091, Staff Nurse, Critical Care  “So with regard to personalities, I think yes, maybe some people might be able to deal with that situation better than how I deal with it.” 0087, Consultant, Critical Care  “I’m not really a stressy person, until it gets to the nitty-gritty, the last minute. It takes quite a fair bit to get me really stressed” 0060, Junior Doctor, Emergency Department  “I think I’m quite, I don’t know what my personality traits came out, but I generally deal with stress quite well, and I am quite relaxed.” 0061, Senior Physiotherapist, Critical Care  “My personal experiences of stress have been to do with feeling as though I’m not doing enough.” 0062, Physiotherapist, Critical Care  “But generally when I’m finding something really, really stressful I go the opposite way to other people. I tend to get really calm” 0068, Staff Nurse, Critical Care  “But I don’t like stress and I don’t like worry or anxiety, so I very quickly recognise when I’m worrying or I’m anxious and will immediately do something about it.” 0071, Sister, Critical Care  “I think they probably go hand-in-hand. Your personality type and how you handle stress.” 0079, Junior Doctor, Critical Care  “I do, because we were all in a similar environment and people deal with it in different ways, and I don’t know if personality and mindset are the same thing, I don’t know, but the way I look at things, I’m like if I get stressed I can’t do my job best that I could do it. So, I always try and make a conscious effort to keep myself calm and then I feel like I can do my own job better. So, I imagine it must be personality.” 0048, Senior Staff Nurse, Critical Care  “I get stressed. I just don’t think about it. I tend to just deal with it, I don’t necessarily overthink it, because it’s part of life and you just have to overcome them, learn, and move on.” 0055, Sister, Critical Care  “I’ve never thought of myself as someone who gets particularly worried about stuff” 0034, Junior Doctor, Emergency Medicine |
| Leadership | Influence on leadership qualities | “So I think it was flexibility between leadership and teamwork. And we all seemed to click and understand each other’s personalities really well in the team. But I think I did definitely see situations where there were a few really strong members who weren’t the leader in the team, or wanted to have their views heard and almost take control a bit more. Which I think is really hard to then get the balance between taking control and then being a leader, so listening to other people’s opinions, and actually understanding what everyone’s role is.” 0057, Physiotherapist, Critical Care  “if you’ve got someone that’s calm and like it’s going to be fine, you’re going to be okay, I’m here if you need anything just let me know, that tends to make them feel instantly relaxed for the shift.” 0072, Sister, Critical Care  “I think one aspect of it is confidence, and calmness, and certainty, and I think, if the leader provides that, I think that’s good for a team because they feel reassured about the plans, in that we’re going in the right direction.” 0075, Physiotherapist, Critical Care  “I’ve definitely seen examples of beneficial traits. Someone that can place themselves very easily in a position of leadership and authority. If you’ve got an emergency situation, you do need someone to take the lead and take people’s first names. Ask them to do specific jobs. Put people into different roles. Lead the team. I think that’s all very beneficial.” 0079, Junior Doctor, Critical Care  “Yes, I think enormously so. Some people internalise it. Some people are going to some people, feel it, and go back over it. Other people have a much more sort of instrumental approach to it, if you like. They say these are the problems I can solve. These are the things I can’t solve, and I’m not going worry about the things that I can’t solve” 0053, Consultant, Critical Care  “I can see, in some of my senior colleagues, where their ability to accept something they can’t change is much better than mine.” 0077, Consultant, Critical Care  “Some consultants are very laid-back and let… Which I find really motivating, that I can take over and run the department. And some consultants like to micromanage everything a registrar does and that creates more stress for a lot of people, actually” 0039, Junior Doctor, Emergency Department  “I like to take people with my personality, the leadership style, I want to have control of everything” 0076, Junior Doctor, Emergency Department  “I have a very collaborative way, that’s my leadership style.” 0087, Consultant, Critical Care  “I’ve always had people, and trainees especially, coming to me and sharing what bothers them, even during pandemic. And I’m always very supportive of them, and I will do what I can to help them.” 0087, Consultant, Critical Care  “But I think I did definitely see situations where there were a few really strong members who weren’t the leader in the team, or wanted to have their views heard and almost take control a bit more. Which I think is really hard to then get the balance between taking control and then being a leader, so listening to other people’s opinions, and actually understanding what everyone’s role is.” 0057, Physiotherapist, Critical Care  “I think one aspect of it is confidence, and calmness, and certainty, and I think, if the leader provides that, I think that’s good for a team because they feel reassured about the plans, in that we’re going in the right direction.” 0075, Physiotherapist, Critical Care  “Very dependent on the senior, really dependent on the senior. I think the most experienced consultants just got on with it. I think the most junior consultants were the ones who tended to be more stressed by it, and you could tell they were more stressed by it.” 0034, Junior Doctor, Emergency Medicine |
|  | Supporting different personalities within the team | “because you’re such a big group of people you will get personalities that you not necessarily clash with, but you have to be a little bit more patient and diplomatic sometimes.” 0080 Staff Nurse, Critical Care  “Lots and lots of people take things home with them and stress out. I see a lot of people doing it and generally, you just need to remind them that they’re seniors and their colleagues are qualified and the patients will still be safe if you go home, as long as you hand over.” 0067, Junior Doctor, Critical Care  “they need more support definitely….because they often feel that they’re letting the team down, because they’re quite slow, they don’t see many patients. It’s not their fault, but it’s just how it is.” 0035, Consultant, Emergency Department  “I think that plays a big part in that, because you know when somebody’s just a bit off or they’re just not their normal selves. And I have known, that some people are more in tune to picking that up than other people are.” 0060, Junior Doctor, Emergency Department  “Or, rather than actually just trying to be empathetic and thinking… Why is this happening, and why is this person not doing the right thing? Or isn’t doing what I think would be the right thing, and trying to understand why. And then work towards that person understanding it, and therefore doing it better.” 0093, Senior Staff Nurse, Critical Care |
