# Supplementary material for: The influence of personality on psychological safety, the presence of stress and chosen professional roles in the healthcare environment
Source: PLoS One. 2023 Jun 5;18(6):e0286796. doi: 10.1371/journal.pone.0286796 (PMC10241408; doi:10.1371/journal.pone.0286796)
Supplement: Attachment — Submitted filename: Response to reviewers.docx [file pone.0286796.s006.docx]

Response to reviewers

**We would like to thank the reviewers for their kind and thorough review of this manuscript. The minor changes have been made within the manuscript, with no alteration to the previous analyses or conclusions drawn. Please find our point-by-point response in line below.**

Reviewer #1: Thanks for the opportunity to review this important paper. The improvement of understanding of team dynamic and contributing factors is vital to improving service delivery and staff well-being. My suggestions to improve aspects of your paper are minor.

**Thank you.**

On page 13, you introduce the 63 participants, and follow immediately with Table 1 which only includes the 58 participants who I assume completed the structured interview. I found this order a little incongruent. The situation can be easily rectified by taking out the line: "The demographics of participants can be viewed in Table 1." from where it is, and after the line: "All 63 completed the 16PF personality assessment and 58 went on to participate in a semi-structured qualitative interview." inserting the line "The demographics of these 58 participants can be viewed in Table 1."

**Thank you for highlighting that this requires clarification. These changes have been made.**

At the end of page 13, there is an apostrophe missing from "A participant's position ...".

**This has been corrected, thank you.**

Similarly, On page 26 "... explore each participant's lived experience ..." needs an apostrophe.

**This has been amended, thank you.**

Also on page 19: "Many participants perceived and individual worker's personality ... " , worker's needs an apostrophe.

**Thank you, this has been corrected.**

In the direct quote on page 20, "I think generally ITU nurses ..." Should this be ICU nurses?

**The participants interchangeably use ICU and ITU to describe the critical care department. For consistency within this manuscript, this has now been edited to read ICU, with no alteration in meaning of the quote.**

On page 28: "The number of nurses recruited from the emergency department was low in number". I think the sentence could be ended at "low" rather than having the "in number" at the end.

**Thank you, this has been amended.**

Finally, the heading to Figure 4 ends with "(All participants, N=58)" Perhaps this should be "(Participants contributing both quantitative and qualitative data, N=58)"

**Thank you, this has now been clarified within the manuscript.**

Other than these small comments, I feel the article reads clearly and introduces valuable findings.

Reviewer #2: Thanks for the revisions. They have addressed my questions and, I believe, strengthened the paper. I noticed one typo: page 17. Add an apostrophe to participants perceptions [ie participants' perceptions].

**Thank you. This has now been addressed.**
